# Public health campaigns and obesity - a critique

**DOI:** 10.1186/1471-2458-11-136

**Published:** 2011-02-27

**Authors:** Helen L Walls, Anna Peeters, Joseph Proietto, John J McNeil

**Affiliations:** 1 Department of Epidemiology & Preventive Medicine, Monash University, The Alfred Centre, 99 Commercial Road, Victoria 3004, Australia; 2 Repatriation Hospital, The Department of Medicine at Austine Hospital, Heidelberg, Victoria 3084, Australia

## Abstract

**Background:**

Controlling obesity has become one of the highest priorities for public health practitioners in developed countries. In the absence of safe, effective and widely accessible high-risk approaches (e.g. drugs and surgery) attention has focussed on community-based approaches and social marketing campaigns as the most appropriate form of intervention. However there is limited evidence in support of substantial effectiveness of such interventions.

**Discussion:**

To date there is little evidence that community-based interventions and social marketing campaigns specifically targeting obesity provide substantial or lasting benefit. Concerns have been raised about potential negative effects created by a focus of these interventions on body shape and size, and of the associated media targeting of obesity.

**Summary:**

A more appropriate strategy would be to enact high-level policy and legislative changes to alter the obesogenic environments in which we live by providing incentives for healthy eating and increased levels of physical activity. Research is also needed to improve treatments available for individuals already obese.

## Background

The increasing prevalence of obesity is now the target of public health effort in most developed countries [1]. The cause of this increasing prevalence of obesity is attributed to societal changes leading to reduced physical activity and increased consumption of energy-dense foods [2,3]. Obesity-reduction strategies in the form of community-based interventions and social marketing campaigns have been established often emphasising the desirability of an ideal body weight. The strategy for achieving this is by eating less, eating healthier foods, and exercising more [4-6] but the primary focus is the maintenance of healthy body weight and shape [7-12]. In general these interventions have had a whole-population focus [2,12-14].

## Discussion

### Community-based interventions and social marketing campaigns for obesity reduction

Community-based interventions are strategies that engage with whole 'communities', conceptualised along geographic boundaries (eg. villages and suburbs) or small social units (e.g. schools and workplaces) in order to address the factors that contribute to an outcome such as weight gain [15]. Examples of such interventions include:

• the building of sporting facilities and playgrounds, mapping out of walking itineraries, and the hiring of sports instructors;

• the offer of cooking classes to families, the offer to 'at risk' families of counselling and overweight children encouraged to see a doctor [16,17];

• changes to canteen menus, the introduction of fruit to canteen menus, reductions in television watching and increases in physical activity after school [18].

In this article we differentiate between these type of small- scale interventions and regulatory interventions that are enacted at governmental level and have wider reach and scope [19]. However the distinction is not always so clear -community-based interventions can utilise policy change, at a local level, to address obesity. The *Recommended Community Strategies and Measurements to Prevent Obesity in the United States: Implementation and Measurement Guide *is an example of a guide for environmental and policy change strategies and measures for local governments and communities [20]. Social marketing is the application of marketing to catalyse behavioural change in a targeted community or population [21].

Most community-based interventions and social marketing campaigns to address obesity have set out to address obesity across the entire community, rather than targeting an obese or overweight subset of the community or population [7,12,13,22-25]. This is understandable, given that weight gain over recent decades has occurred across the range of body weight, not simply in the overweight or obese [26-28]. The focus of social marketing is inherently behaviour change in the individual [13]. Community-based interventions vary in the emphasis placed on individual behavioural change and in their explicit focus on obesity and body image [2,12,22,29,30]. Most social marketing and community-based interventions have emphasised the importance of healthy eating and physical activity, and some have emphasised the desirability of achieving a healthy body weight [4-7,12,28]. Less commonly other factors likely to affect body weight, such as stress and lack of sleep [31-33], are also addressed.

There is sparse evidence that even the most well-designed of such interventions are effective at addressing obesity, either weight gain or maintenance, and virtually none that they are sustainable in the long term [13,25,34-37]. The results of several programmes have been published. The substantially beneficial ones have generally been in children [16,17,38-40], in whom behaviour can be more easily modified than in adults [2,41-44]. Social marketing campaigns that have targeted weight loss explicitly have generally shown poor weight loss outcomes [13,45].

A well recognised potential downside of the community-based programmes and social marketing campaigns targeting obesity is their promotion, exacerbated by the media, of the social desirability of thinness [46,48-50]. The desirability of 'normal' body weight is emphasised to an extent that in some cases overstates the public health evidence for benefit; and ignores ethnic and age differences [51-53]. The reinforcement by such interventions of the already entrenched attitudes regarding the undesirability of being overweight may be harmful to some people [23,34,49,53,54]. The mistreatment of obese people has been well documented [46,53,55-57]. But there is little known about the psychological impact on people who are mild to moderately overweight, particularly in young women and female adolescents, who face the strongest social pressure to be thin [58,59].

### High-risk approaches to obesity reduction

The limited success of community-based programmes and social marketing campaigns is matched by equally serious limitations in the 'high-risk' approach to severely obese patients [34,40,60].

Addressing lifestyle (diet and physical activity) is generally the first approach tried for assisting weight loss in the obese. But such change rarely achieves satisfactory results [34,60,61]. If success is not achieved following lifestyle change, the key methods for reducing weight in obese patients are drug treatment and, in the case of severely obese patients, surgery [60,62,63].

Anti-obesity or weight-loss drugs are those pharmaceutical agents designed to reduce or control weight by altering physiological processes [64]. However the weight loss achievable from such interventions is fairly minimal - approximately 5% of body weight [65-67]. Furthermore, current drug treatments for obesity appear to have little long-term value and are associated with adverse effects [66,68]. Two of the most widely used agents have recently been removed from market because of serious adverse effects [69].

The body responds to the reduced food consumption during weight loss via dieting or medication by implementing compensatory responses with the aim of achieving positive energy balance [70]. Obesity surgery or bariatric surgery works by circumventing these compensatory responses, creating a feeling of satiety after a small intake of food [71], and resulting in the maintenance of a negative energy balance [70]. In contrast to drug therapy, surgery has demonstrated significant efficacy [72-74]. Long-term studies have shown that surgery can result in reversal of type 2 diabetic states, improvement in cardiovascular risk factors, and a significant reduction in mortality [75-78]. But access to this intervention is always likely to be limited to the individuals at the very highest risk and those who can afford the procedure [79,80]. Furthermore, serious adverse effects are experienced by some patients having undergone surgery [74,81].

### Proposed approach

Given the uncertainty of the balance between 'benefit' and harm associated with community-based programmes and social marketing campaigns that specifically target the undesirability of obesity, the approach to controlling the increasing prevalence of this condition should shift towards dietary and physical activity interventions where there is a better established evidence base and a stronger prospect of benefit [2,61,82-88]. This could best be achieved by decreasing the focus on undesirable features of obesity and towards a focus on the public health benefits of healthy diets with a low content of processed, energy-dense foods and a high intake of fruit and vegetables, and physical activity. Such changes should focus on the benefits of a healthy diet and physical activity rather than on obesity per se. However promotion of a healthy diet and increased physical activity would be expected to lead to the achievement of weight control in current generations, and the prevention of weight gain in future generations.

Having said this, community-based interventions designed to improve dietary quality and physical activity levels have generally been unsuccessful whenever they are dependent on an individual acquiring the motivation to eat/act differently to the people in his/her social and peer groups or consume a diet markedly different to that readily available in the community [13,54,89,90]. Strategies reliant on individual behaviour change are unlikely to achieve their goals [91,92]. Success in encouraging consumers to make healthy dietary choices is likely to require society-wide changes that reduce the attractiveness and availability of energy-dense, nutrient-poor foods [93,94]. Healthy options need to be made more accessible, available and desirable than the unhealthy alternatives. It is unlikely that this will be achieved without legislative changes [93].

As a preventive measure, regulatory reform is one of society's most powerful mechanisms for change, with the potential to create significant shifts in culture, attitudes and behaviour. There is currently little evidence in support of a regulatory approach to addressing obesity [95-98]. However this lack of evidence is likely due to the early stage we are at in terms of addressing obesity. Regulation in many other areas of public health - seat belt use, vaccinations and occupational safety, for example - has resulted in important health benefits [95,99,100]. One of history's key regulatory reforms in public health, the 'sanitary reform' of 19^th ^century Britain, has been voted by readers of the *British Medical Journal *as the most important medical milestone since 1840 [101]. To use an example from tobacco control, the marked reductions in the prevalence of smoking observed in most developed countries over recent decades could not have been brought about without regulatory means [102].

Some of these targets for legislative change had advantages over obesity. However advocates of these other areas, advocates of tobacco control, for example, also faced considerable challenges [103]. Given the history of regulatory reform in public health, it is likely that well-designed policy and legislative changes could also play an important role in obesity prevention.

More work is needed to develop the most appropriate framework for such policy and legislative change to improve the nutrition in developed countries. The World Health Organization's *Global Strategy on Diet, Physical Activity and Health *is a guide for developing such a framework [104]. However others have suggested some specific measures, ones predominantly focused on food and nutrition. A three-pronged strategy outlined by Frieden et al. (2010) with which such change could be enacted comprises of:

(1) food pricing adjustments such as subsidies on fruit and vegetables and taxation applied to energy-dense nutrient-poor food;

(2) increasing exposure to healthy food (and decreasing exposure to unhealthy food) via zoning and restrictions on the display of foods in locations such as supermarkets, for example; and

(3) improving the image of healthy food (and making unhealthy food less attractive) via restrictions on advertising and the presentation of caloric contents of restaurant meals, for example [105].

Others have also proposed specific measures similar to the approach outlined above [94,106,107].

The enactment of such policies should be based on a broad, whole-systems approach to food policy and public health [13,108,109]. Such consideration would involve health professionals working with people from outside the health sector and being involved in policy development outside their usual areas of expertise. The specific options cannot generally be tested ahead of implementation; however they are practical, based on reasoned and reasonable assumptions [94], and would be enacted from a whole-systems paradigm. Without such a whole-systems approach to policy change, there is the potential for one policy to negate another's effectiveness [13,28,109]. For example, a system of subsidising fruit and vegetables and increasing taxation on 'unhealthy' foods could be undermined by the strong agricultural subsidies on the production of sugar, meat and dairy products, as reportedly occurs both in the US and EU currently [110,111]. (Others have argued for a negligible effect of such interventions on consumer prices of food [112]).

A regulatory approach to addressing obesity also has an additional potential advantage over community-based and social marketing interventions - a greater potential for reducing inequalities in obesity. The messages espoused by community-based and social marketing interventions are more likely to be heeded by those with already high levels of education; people with lower educational attainment are much less likely to change their behaviour as a result of education efforts [113-117]. Community-based interventions and social marketing campaigns can focus specifically on areas (e.g. schools) with a high density of families of low socio-economic status and poor education [118,119]. However legislative measures, and particularly those broader policies influencing income distribution, employment, housing and social services, are more likely to affect the whole population, regardless of educational attainment [93,120]. Furthermore, Friel et al. (2007) and others have suggested that not just obesity itself, but also its unequal distribution across society, are driven by the same societal conditions [13,97]. Thus regulatory reform addressing these same conditions could be considerably beneficial. Regulatory interventions also have the benefit of less potential to stigmatise obesity.

One of the main difficulties with enacting such policy and legislative change is the opposition from the food and beverage industry [2,121-123]. The industry has strongly opposed legislative and regulatory approaches that encourage healthy eating when these may restrict its profitability [124-126]. It has placed considerable pressure on federal and state legislatures, at least in the United States, to enact statutes prohibiting lawsuits against food and beverage companies and restaurants for obesity-related claims [2,125]. It has supported health promotion measures addressing obesity, but those measures with the likely outcome of increasing consumer confusion rather than promoting healthy eating [93]. The food and beverage industry must be regulated in new ways if any change in the epidemiology of obesity is to be achieved [2,126-128].

Furthermore, the political context in which regulatory change occurs must be better considered and integrated into the strategic planning of the implementation of any chosen framework of regulatory intervention. Analysis of the history of regulatory interventions in public health has revealed the public recognition of a 'crisis' situation as a key factor preceding regulatory intervention [103]. Thus, more effectively structured communication of the evidence regarding the crisis reached in terms of obesity and the influence of the environment on individual attitudes and behaviours in regards to nutrition will be necessary for the generation of the requisite public support [98,100,103]. This information must be sensitively communicated and debated, however, so as to avoid further stigmatising individuals with obesity [98]. The 'individual choice' paradigm must be regularly challenged [103].

In addition to policy and legislative change, further research is required to improve high-risk interventions capable of assisting those with established obesity. Such individuals are unlikely to be helped by population-wide programs [2,34]. Improved high-risk interventions are important to assist the increasingly large proportion of the population in need of medical assistance to induce weight loss [2,34,60,129]. The prevalence of obesity and severe obesity is high in a number of countries. In the US in 2007-08, the prevalence of obesity in adults was 34%. The prevalence of severe obesity - grades 2 and 3 - was 14% and 6%, respectively [130]. Furthermore, Walls et al. (2010) have shown in Australia that if current incidence rates remain the same the prevalence of obesity will increase by 70% between 2000 and 2025. Recent data validates this prediction [131]. Research to improve high-risk interventions is also important considering that even if policy and legislative chances were enacted to combat obesity, it is likely that their positive impact would be in preventing weight gain, and would be most beneficial for the younger generation [34,132].

## Summary

Community-based programmes, social marketing campaigns and associated media focussing on the undesirability of obesity are poorly supported by existing evidence, and have the potential for harm.

A more fruitful area for intervention is the enactment of high-level policy and legislative changes to provide incentives for healthy eating and increased physical activity. Such change must impact on the ability of the food and beverage industry to encourage unhealthy consumption. Adoption of healthier eating habits, complemented with increased levels of physical activity, provides the population-wide strategy most likely to reduce the incidence of obesity.

The development of evidence for regulatory reform addressing obesity should be a priority. Further research is also needed to improve management options for those with established obesity who are unlikely to benefit from population-wide approaches.

## Competing interests

JP is the Chair of the Optifast Medical Advisory Board for Nestle Australia. JJM and JP were past members of the Medical Advisory Board for Sibutramine for Abbott.

## Authors' contributions

HLW wrote the manuscript. All authors contributed conceptually to the article and reviewed drafts of the manuscript.

## Pre-publication history

The pre-publication history for this paper can be accessed here:

http://www.biomedcentral.com/1471-2458/11/136/prepub
